# Human Thalamic-Prefrontal Peduncle Connectivity Revealed by Diffusion Spectrum Imaging Fiber Tracking

**DOI:** 10.3389/fnana.2018.00024

**Published:** 2018-04-17

**Authors:** Chuanqi Sun, Yibao Wang, Run Cui, Chong Wu, Xinguo Li, Yue Bao, Yong Wang

**Affiliations:** ^1^Department of Innovative Medical Photonics, Preeminent Medical Photonics Education & Research Center, Institute for Photonics Research, Hamamatsu University School of Medicine, Hamamatsu, Japan; ^2^Department of Neurosurgery, First Affiliated Hospital of China Medical University, Shenyang, China; ^3^Department of Neurosurgery, Anshan Central Hospital, Anshan, China

**Keywords:** thalamic-prefrontal peduncle, diffusion spectrum imaging, fiber tractography, connectivity pattern, white matter

## Abstract

The thalamic-prefrontal peduncle (TPP) is a large bundle connecting the thalamus and prefrontal cortex. The definitive structure and function of the TPP are still controversial. To investigate the connectivity and segmentation patterns of the TPP, we employed diffusion spectrum imaging with generalized q-sampling reconstruction to perform both subject-specific and template-based analyses. Our results confirmed the trajectory and spatial relationship of the TPP in the human brain and identified the connection areas in the prefrontal cortex. The TPP-connecting areas identified based on Brodmann areas (BAs) were BAs 8–11 and 45–47. Based on the automated anatomical atlas, these areas were the medial superior frontal gyrus, superior frontal gyrus, middle frontal gyrus, pars triangularis, pars orbitalis, anterior orbital gyrus, and lateral orbital gyrus. In addition, we identified the TPP connection areas in the thalamus, including the anterior and medial nuclei, and the lateral dorsal/lateral posterior nuclei. TPP fibers connected the thalamus with the ipsilateral prefrontal BAs 11, 47, 10, 46, 45, 9, and 8 seriatim from medial to lateral, layer by layer. Our results provide further details of the thalamic-prefrontal peduncle structure, and may aid future studies and a better understanding of the functional roles of the TPP in the human brain.

## Introduction

Thalamic peduncles, or radiations, are a large bundle of fan-like fibers connecting the thalamus and cortex, and well-described in both human and monkey brains. Based on the connected cortical area, thalamic peduncles are commonly divided into five components: the anterior, superior, lateral (or extracapsular), posterior, and inferior peduncles (de Castro et al., 2005; Schmahmann and Pandya, 2006; Serra et al., 2017). A classic neuroanatomy study using white matter fiber microdissection defined the fibers originating in the thalamus and radiating along the anterior limb of the internal capsule toward the prefrontal cortex as the anterior thalamic peduncle (ATP), and the fibers radiating from the thalamic pulvinar to the tip of the temporal lobe as the inferior thalamic peduncle (ITP) (Serra et al., 2017). However, a recent autoradiography study in monkeys, as well as human studies, reported that the ITP also contains a large bundle of fibers extending to the orbital cortex (Schmahmann and Pandya, 2006; Jiménez et al., 2007, 2013). To avoid confusion, we consider all fibers connecting the thalamus and prefrontal cortex as one fiber bundle, termed the thalamic-prefrontal peduncle (TPP) and composed of the ATP and parts of the ITP.

In recent years, diffusion imaging techniques, such as diffusion tensor imaging (DTI) and improved high-definition fiber tracking (HDFT), have provided non-invasive methods for investigating brain white matter fibers and connectivity *in vivo* (Basser et al., 2000; Fernandez-Miranda et al., 2012). Previous DTI studies have delineated the trajectories of the thalamic peduncles. The fibers originate from the thalamus and project to the brain cortex through the internal capsule. Although DTI reveals fiber trajectory, it does not provide detailed information on the connectivity patterns and precise termination areas of the thalamic peduncles (Wakana et al., 2004). An HDFT study tracking white matter fibers and resolving complex fiber crossings described the thalamocortical projection system (Fernandez-Miranda et al., 2012). The study observed the thalamic fibers radiating to the frontal, central, parietal, and occipital cortical areas, and identified the origin/termination areas of the fibers in the thalamus: the medial and anterior portions of the thalamus connected to the orbitofrontal cortex, the ventral anterior and lateral potions to the prefrontal cortex, and most of the posterior potion to the parieto-occipital cortex. However, this study still did not reveal the precise connected cortical regions of the fibers (Fernandez-Miranda et al., 2012). Another study, based on probabilistic tractography algorithms, showed similar results to those obtained with HDFT; however, it also failed to identify the detailed cortical connections of the thalamic peduncles. The study treated the entire prefrontal cortex as a single region, without subdivision, and only characterized the connections between the thalamic mediodorsal and ventral anterior nuclei, and the prefrontal cortex (Behrens et al., 2003).

The existence of the TPP has been established in classic neuroanatomy, autoradiography, and diffusion imaging studies of monkey and human brains. However, the detailed TPP structure and connectivity patterns, such as specific terminal areas in the prefrontal cortex, remain unclear. In the present study, we aimed to identify the connectivity patterns of the human TPP. To overcome the limitations of DTI studies (Alexander and Barker, 2005; Le Bihan et al., 2006), we employed a high-resolution form of white matter tractography, diffusion spectrum imaging (DSI) (Wedeen et al., 2005, 2008), reconstructed by generalized q-sampling imaging (GQI) (Yeh et al., 2010; Zhang et al., 2013), to sufficiently resolve the complex fiber crossings involved in the TPP.

## Materials and methods

### Participants

Nine neurologically healthy adults (4 males; all right-handed; age range 22–34) took part in this experiment. All participants were prescreened prior to testing to rule out any contraindications to MR imaging. Written informed consent was obtained from all participants prior to testing. The procedures used were approved by the institutional review board, including the ethics committee of China Medical University.

### Imaging acquisition and reconstruction

DSI data were acquired on a 3T Tim Trio System (Siemens) with a 32-channel head coil. A head stabilizer was used to prevent head motion. Image acquisition involved a 43 min, 257-direction scan using a twice-refocused spin-echo echo-planar imaging sequence and multiple *q*-values (echo time [TE] = 157 ms, repetition time [TR] = 9,916 ms, field of view [FoV] = 231 × 231 mm, voxel size = 2.4 × 2.4 × 2.4 mm, b_max_ = 7000 s/mm^2^) (Wedeen et al., 2005). We also included high-resolution anatomical imaging for anatomical comparisons, employing a 9 min T1-weighted axial magnetization prepared rapid gradient echo (MPRAGE) sequence (TE = 2.63 ms, TR = 2,110 ms, flip angle = 8°, 176 slices, voxel size = 0.5 × 0.5 × 1.0 mm, FoV = 256 × 256 mm). DSI data were reconstructed with a generalized Q-sampling imaging approach (Yeh et al., 2010). The orientation distribution functions (ODFs) were reconstructed to 362 discrete sampling directions and a mean diffusion distance of 1.2 mm.

### Human connectome projection (HCP)-842 template

In addition to subject-specific analyses, we conducted fiber tracking on a publicly available high-definition fiber tracking template (HCP-842 atlas). The data were averaged from a total of 842 subjects included in the HCP dataset maintained by the WU-Minn HCP Consortium (2015, 900-subject release) and distributed under the WU-Minn HCP open access data use terms (Van Essen et al., 2013). Diffusion images were acquired using a multishell diffusion scheme. The b-values were 1000, 2000, and 3000 s/mm^2^, with 90 diffusion sampling directions in each case. The in-plane resolution was 1.25 mm, and the slice thickness was 1.25 mm. The diffusion data were reconstructed in the MNI space using generalized q-space reconstruction (Yeh and Tseng, 2011) to obtain the spin distribution function. A diffusion sampling length ratio of 1.25 was used, and the output resolution was 2 mm. The atlas was constructed by averaging the SDFs of 842 individual subjects. The HCP-842 template is available freely for download at http://dsi-studio.labsolver.org.

### Fiber tracking and analysis

The DSI Studio software, an open-source diffusion algorithm analysis tool freely downloadable at http://dsi-studio.labsolver.org, was used for fiber tracking. Rather than adopt a whole-brain fiber-tracking procedure, we chose to use an approach based on ODF-streamlined regions of interest (ROIs) (Yeh et al., 2010). In voxels with multiple fiber orientations, fiber tracking was initiated separately for each orientation, and fiber progression continued with a step size of 1.2 mm (subjects) or 1 mm (template; half the spatial resolution), a minimum fiber length of 20 mm, and a turning angle threshold of 60°. If multiple fiber orientations existed in the current progression location, the fiber orientation nearest to the incoming direction and forming a turning angle smaller than 60° was selected to determine the next moving direction. To smoothen each track, the next moving directional estimate of each voxel was weighted by 20% of the previous incoming direction and 80% of the nearest fiber orientation (Wang et al., 2013; Fernández-Miranda et al., 2015). This progression was repeated until the quantitative anisotropy (QA) (Yeh et al., 2010) of the fiber orientation dropped below a preset threshold (0.03–0.06 depending on the subject) or until no fiber was selected within the 60° angular range in the progression. The tracking was stopped when it had generated 40,000 fiber tracts.

**Figure 1:**
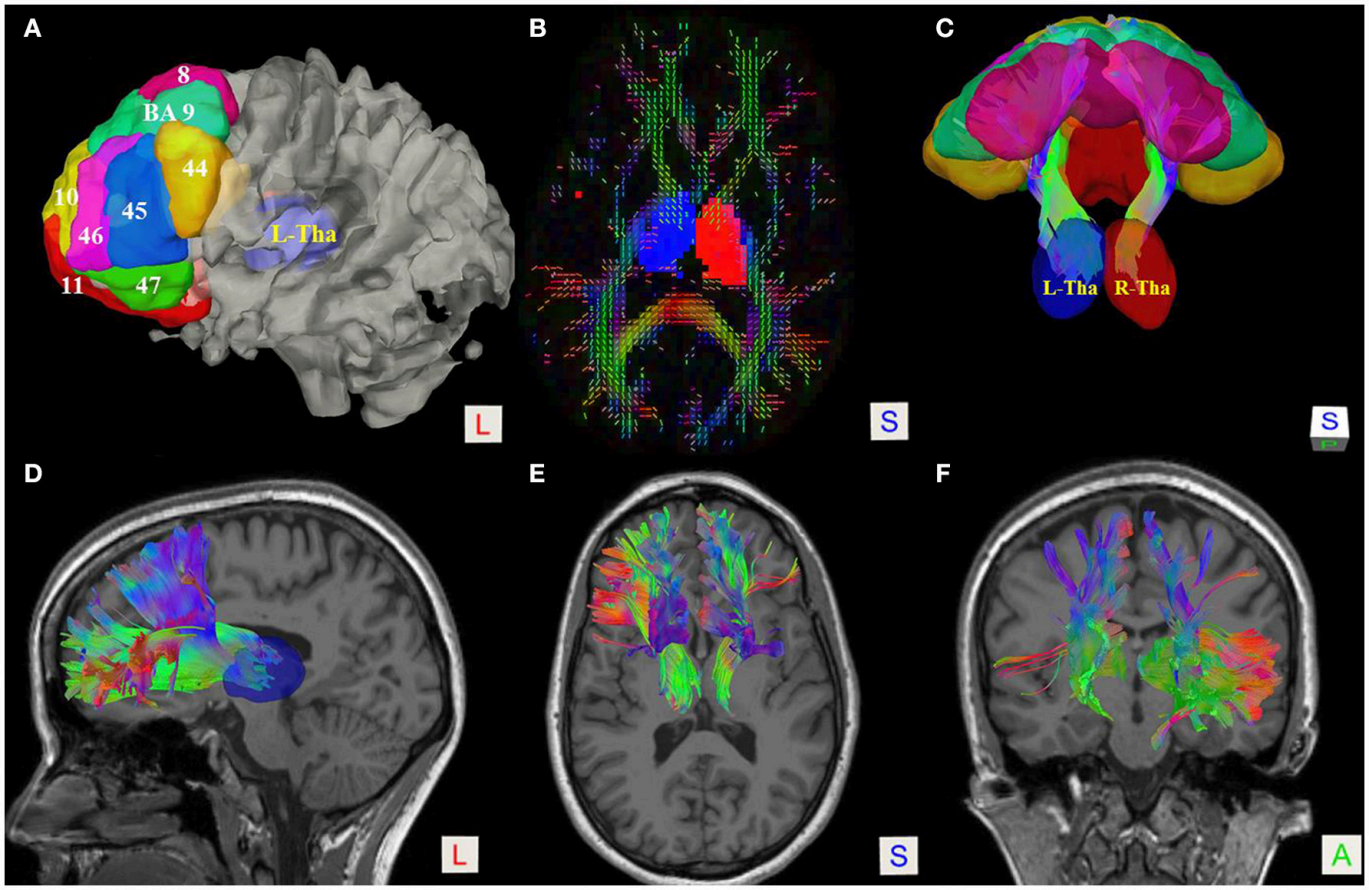
Fiber tractography of the thalamic-prefrontal peduncle (TPP; subject No. 8). **(A)** Seeding regions of interest (ROIs) were placed on the prefrontal cortex, including Brodmann areas (BAs) 8–11 and 44–47. The thalamus (Tha) was set as the ending ROI. The ROIs were overlaid on the white-matter surface. **(B)** Axial slice at the thalamus level on a quantitative anisotropy (QA) color map; Ending ROIs were placed at left thalamus (blue) and right thalamus (red). **(C)** Posterior-superior view of the TPP and ROIs. **(D–F)** Lateral, superior, and anterior views of the TPP, overlaid on T1 MRI slices.

To reconstruct the right and left TPP, we used bilateral prefrontal cortical ROIs, including Brodmann areas (BAs) 8–11 and 44–47, as seeding regions (Figure 1). Another ROI was set at the left or right thalamus as an end region. To quantify and compare the right and left sides, as well as the contribution of each seeding region, we counted the numbers of voxels occupied by the fiber trajectories (streamlines). In addition, to understand the spatial relationship of the TPP with adjacent fiber tracts, the inferior frontal-occipital fasciculus (IFOF), anterior part of the corpus callosum (A-CC), and corticospinal tracts (Cort-Sp) were reconstructed using the same fiber tracking parameters as in the TPP reconstruction.

To determine the cortical terminations and segmentation of the TPP, we imported cortical prefrontal areas from two different atlases, the BA and improved automated anatomical (AAL2) atlases (Tzourio-Mazoyer et al., 2002; Rolls et al., 2015). DSI Studio used linear transformations to register both atlases on a subject's diffusion space.

### Statistical analysis

Statistical analyses were carried out using SPSS 22.0 (SPSS, Chicago, IL). *T*-tests were used to evaluate parametric differences. *P* ≤ 0.05 was considered to be statistically significant. Continuous variables are presented as means ± standard deviation.

## Results

### Human TPP trajectory

**Figure 2:**
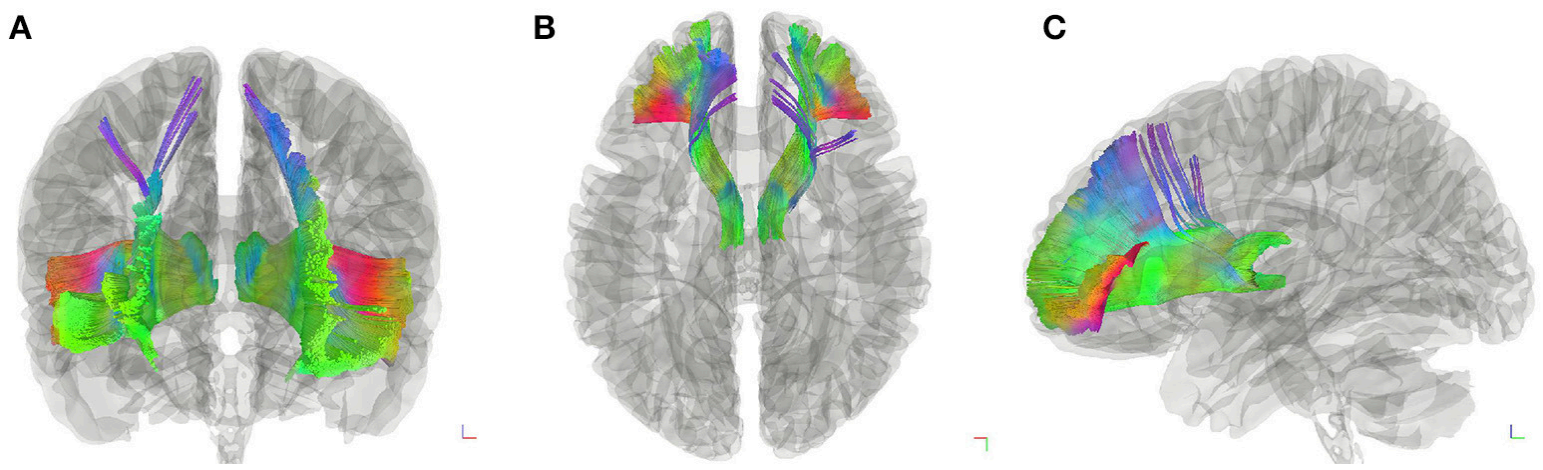
Fiber tractography of the thalamic-prefrontal peduncle (TPP; HCP-842 template). Anterior **(A)**, superior **(B)**, and lateral **(C)** views show the trajectories of the left and right TPP in the HCP-842 template, which represents the average of 842 subjects.

**Figure 3:**
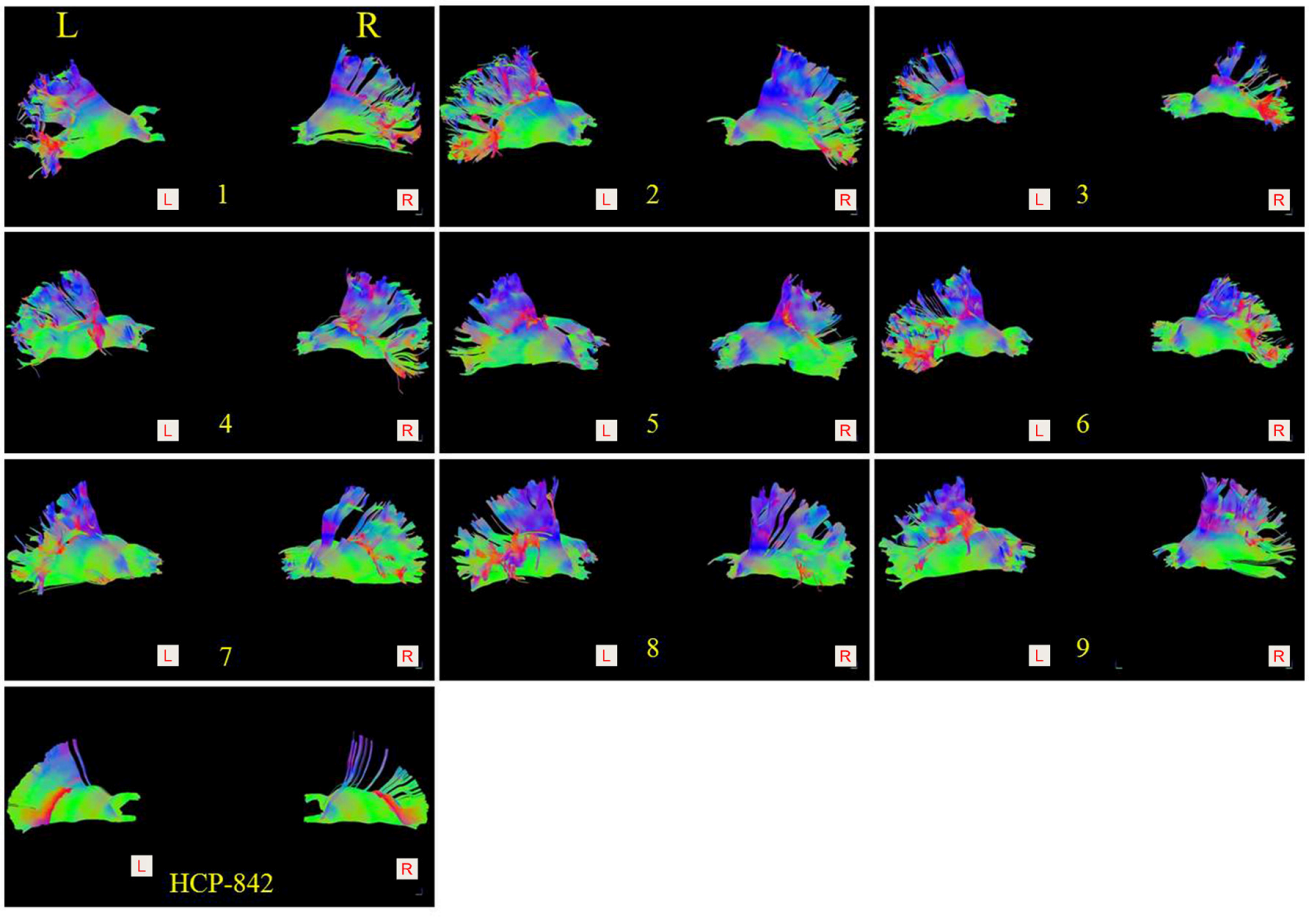
Comparison of the thalamic-prefrontal peduncles (TPPs) between 9 subjects (subject: 1–9) and the HCP-842 template. Both the left (**Left lateral view**) and right (**Right lateral view**) TPP of each subject are presented. Similar trajectories were observed in 20 hemispheres (18 hemispheres from 9 subjects; 2 hemispheres from the HCP-842 template).

Subject-specific fiber tractography consistently showed a large bundle of fibers projecting anteriorly from the thalamus toward the prefrontal lobe, which included most of the selected seeding regions (Figures 1C–F). Template (HCP-842) tractography showed similar results (Figure 2). This distinct group of fibers originated from the thalamus and projected along the anterior limb of the internal capsule to the prefrontal cortex, primarily including the superior frontal gyrus (SFG), orbital-frontal cortex (OFC), and inferior frontal gyrus (IFG). A qualitative study of 20 hemispheres (18 hemispheres from 9 subjects and 2 hemispheres from the HCP-842 template) showed nearly identical trajectories (Figure 3).

### Spatial relationship of the TPP with adjacent fiber tracts and structures

To visualize the anatomical spatial relationship of the TPP with adjacent tracts, the Cort-Sp, IFOF, and A-CC were reconstructed using *in vivo* fiber tractography.

**Figure 4:**
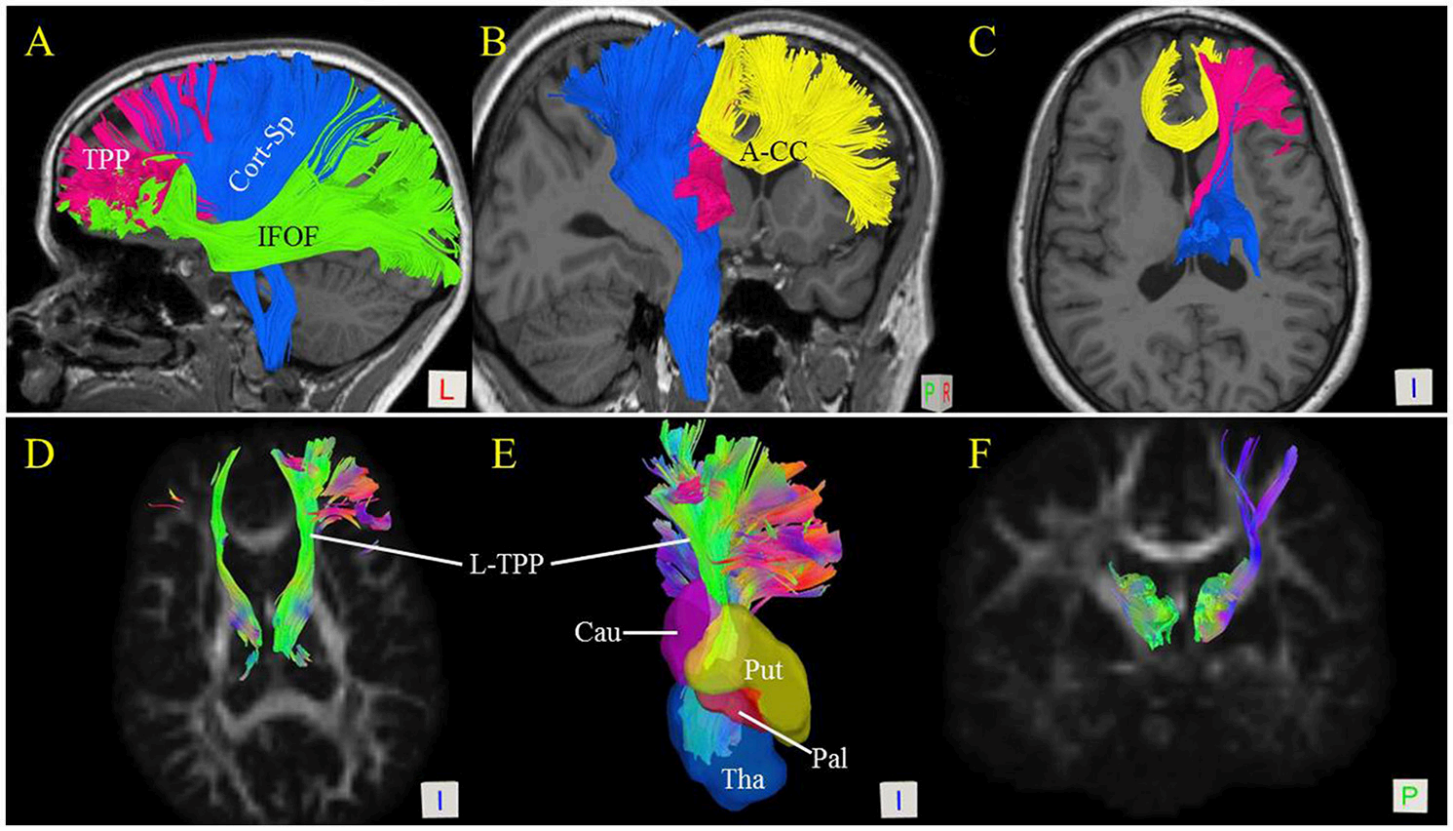
Spatial relationship of the thalamic-prefrontal peduncle (TPP) with adjacent fiber tracts and structures. **(A)** Lateral view shows the spatial relationships of the left TPP (red) with the inferior frontal-occipital fasciculus (IFOF; green) and corticospinal tracts (Cort-Sp; blue). **(B)** Posterior-medial view of the left TPP, Cort-Sp, and anterior corpus callosum (A-CC; yellow). **(C)** Inferior view of the TPP, Cort-Sp, and A-CC. **(D)** Inferior view of the bilateral TPP shows the fiber bundles projecting to the prefrontal cortex through the anterior limb of the internal capsule. **(E)** Inferior view reveals the spatial relationships of the TPP with the thalamus (Tha), and caudate (Cau), putamen (Put), and pallidum (Pal) nuclei. **(F)** Posterior view of the bilateral TPP.

The Cort-Sp has been well-described and is situated superficially to the TPP. In our analyses, the main trunk of the TPP was located medially to the Cort-Sp tract and projected anteriorly to the prefrontal cortex (Figures 4A–C). The IFOF is a large bundle of fibers that tracks from the prefrontal region to the occipital region via the ventral part of the external/extreme capsule. The prefrontal part of the IFOF was situated immediately ventrally and superficially to the prefrontal part of the TPP fibers (Figure 4A). Importantly, the interconnected prefrontal cortex regions of the TPP and IFOF overlapped partially at the IFG and orbital gyrus. The corpus callosum (CC) is a large bundle of commissural fibers connecting the left and right brain hemispheres. The A-CC was located medially to the TPP (Figures 4B,C).

The Free Surfer parcellations (Desikan et al., 2006) of the caudate, putamen, and pallidum nuclei were overlaid on the TPP (Figure 4E). Our results showed the TPP pathway to be adjacent to the head of the caudate nucleus, the putamen, and the pallidum nucleus. The fiber bundles projected to the prefrontal cortex through the anterior limb of the internal capsule (Figures 4C–F).

### TPP segmentation and connectivity

**Figure 5:**
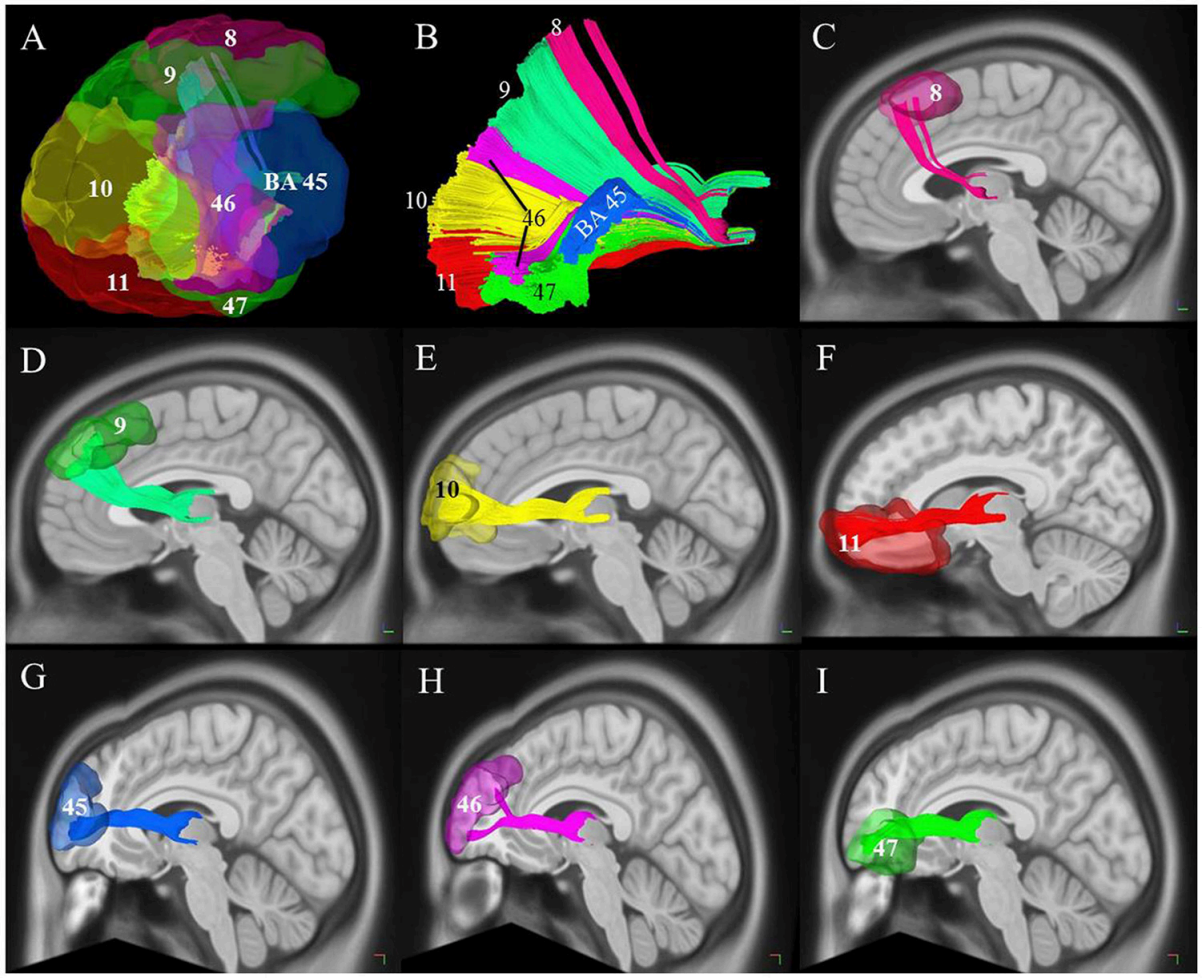
Cortical connectivity and segmentation of the thalamic-prefrontal peduncle (TPP) based on the Brodmann atlas (HCP-842 template). **(A)** The TPP-connected areas in the prefrontal cortex included Brodmann areas (BAs) 8, 9, 10, 11, 45, 46, and 47. **(B)** Segmentation of the TPP fibers based on prefrontal BAs. Different fibers are assigned different colors. **(C–I)** Views of each tract based on the segmentation. Fibers of each segmentation are colored according to **(A,B)**.

To investigate the connections of the TPP fibers between the prefrontal cortex and thalamus, we identified different prefrontal cortical regions based on BAs and the AAL2 atlas. The areas connected to the prefrontal cortex primarily included BAs 8, 9, 10, 11, 45, 46, and 47 (Figure 5). TPP fibers terminated bilaterally at BAs 9, 10, and 11 in all 9 subjects. Fibers converged bilaterally at BAs 8, 45, and 46 in 8 out of 9 subjects. Fibers terminated at BA 47 on the left side in 7, and on the right side in 6 out of 9 subjects. Additionally, some fibers terminated at BA 44 on the left side in only 2, and on the right side in only 1 out of 9 subjects. Imaging using the HCP-842 template showed similar results (Table 1, Figure 5). TPP segmentation based on BAs is shown in Figure 5.

**Table 1:** Quantitative study of the TPP.

	**L**	**R**	**BA 8**		**BA 9**		**BA 10**		**BA 11**		**BA 44**		**BA 45**		**BA 46**		**BA 47**	
			**L**	**R**	**L**	**R**	**L**	**R**	**L**	**R**	**L**	**R**	**L**	**R**	**L**	**R**	**L**	**R**
1	32.95	29.86	5.91	4.56	6.79	5.15	6.65	8.54	2.72	2.61	0	0	0	0.96	4.83	2	6.05	6.04
	(52.5)	(47.5)	(9.4)	(7.3)	(10.8)	(8.2)	(10.6)	(13.6)	(4.3)	(4.2)				(1.5)	(7.7)	(3.2)	(9.7)	(9.5)
2	49.46	33.13	9.19	8.09	7.5	6.57	9.42	8.55	4.51	3.86	1.95	0	2.76	0	8.11	4.32	6.02	1.74
	(59.9)	(40.1)	(11.1)	(9.8)	(9.1)	(8.0)	(11.4)	(10.3)	(5.5)	(4.7)	(2.4)		(3.3)		(9.8)	(5.2)	(7.3)	(2.1)
3	39.3	34.6	3.46	4.75	4.79	1.69	9.11	7.06	14.4	7.48	0	0	1.7	5.18	3.56	2.56	2.28	5.88
	(53.2)	(46.8)	(4.7)	(6.4)	(6.5)	(2.3)	(12.3)	(9.5)	(19.5)	(10.1)			(2.3)	(7.0)	(4.8)	(3.5)	(3.1)	(8.0)
4	36.46	36.37	4.79	5.47	7.46	7.93	10.32	8.92	1.79	2.12	0.93	2.07	5.09	1.94	6.08	6.16	0	1.76
	(50.1)	(49.9)	(6.6)	(7.5)	(10.2)	(10.9)	(14.2)	(12.2)	(2.5)	(2.9)	(1.3)	(2.8)	(7.0)	(2.7)	(8.3)	(8.5)		(2.4)
5	31.68	29.22	0	0	8.45	7.47	9.26	8.82	5.09	10.38	0	0	3.24	2.55	0	0	5.64	0
	(52.0)	(48.0)			(13.9)	(12.3)	(15.2)	(14.5)	(8.3)	(17.0)			(5.3)	(4.2)			(9.3)	
6	46.59	42.27	7.97	10.27	5.25	5.74	10.73	3.92	7.16	5.65	0	0	4.65	4.28	5.63	8.08	5.2	4.33
	(52.4)	(47.6)	(9.0)	(11.6)	(5.9)	(6.5)	(12.1)	(4.4)	(8.1)	(6.3)			(5.2)	(4.8)	(6.3)	(9.1)	(5.8)	(4.9)
7	30.3	33.48	3.81	4.8	5.26	3.63	5.24	8.48	5.36	7.03	0	0	3.91	2.66	3.12	4.66	3.6	2.22
	(47.5)	(52.5)	(6.0)	(7.5)	(8.2)	(5.7)	(8.2)	(13.3)	(8.4)	(11.0)			(6.1)	(4.2)	(4.9)	(7.3)	(5.7)	(3.5)
8	37.44	25.27	3.47	5.14	5.3	4.99	5.87	8.33	6.4	3.48	0	0	7.54	1.85	6.08	1.48	2.78	0
	(59.7)	(40.3)	(5.5)	(8.2)	(8.5)	(7.9)	(9.4)	(13.3)	(10.2)	(5.5)			(12.0)	(3.0)	(9.7)	(2.4)	(4.4)	
9	33.7	26.36	4.12	3.45	6.33	5.97	7.91	6.25	5.8	4.94	0	0	8.77	2.45	0.77	3.3	0	0
	(56.1)	(43.9)	(6.9)	(5.7)	(10.5)	(10.0)	(13.2)	(10.4)	(9.6)	(8.2)			(14.6)	(4.1)	(1.3)	(5.5)		
Mean	37.54	32.28	4.75	5.17	6.35	5.46	8.28	7.65	5.91	5.28	—	—	4.18	2.43	4.24	3.62	3.51	2.44
	(53.7)	(46.3)	(6.6)	(7.1)	(9.3)	(8.0)	(11.8)	(11.3)	(8.5)	(7.8)			(6.2)	(3.5)	(5.9)	(5.0)	(5.0)	(3.4)
Template	22.83	20.99	1.55	1.33	3.54	1.91	3.78	4.88	3.7	4.35	0	0	3.25	1.9	2.63	2.34	4.38	4.28
	(52.1)	(47.9)	(3.5)	(3.1)	(8.1)	(4.4)	(8.6)	(11.1)	(8.5)	(9.9)			(7.4)	(4.3)	(6.0)	(5.3)	(10.0)	(9.8)

*The tract (and sub-tract) volumes were counted by the number of voxels occupied by the fiber trajectories (streamlines) and are reported in milliliters (ml). The figures in parentheses represent the relative volume of each tract and subtract in percentages (%), where the left and right TPP together represent 100% of the volume. BA, Brodmann Area. “—” indicates that the mean of the tract volumes of BA 44 were not calculated because only three out of 18 hemispheres showed connected tracts*.

**Figure 6:**
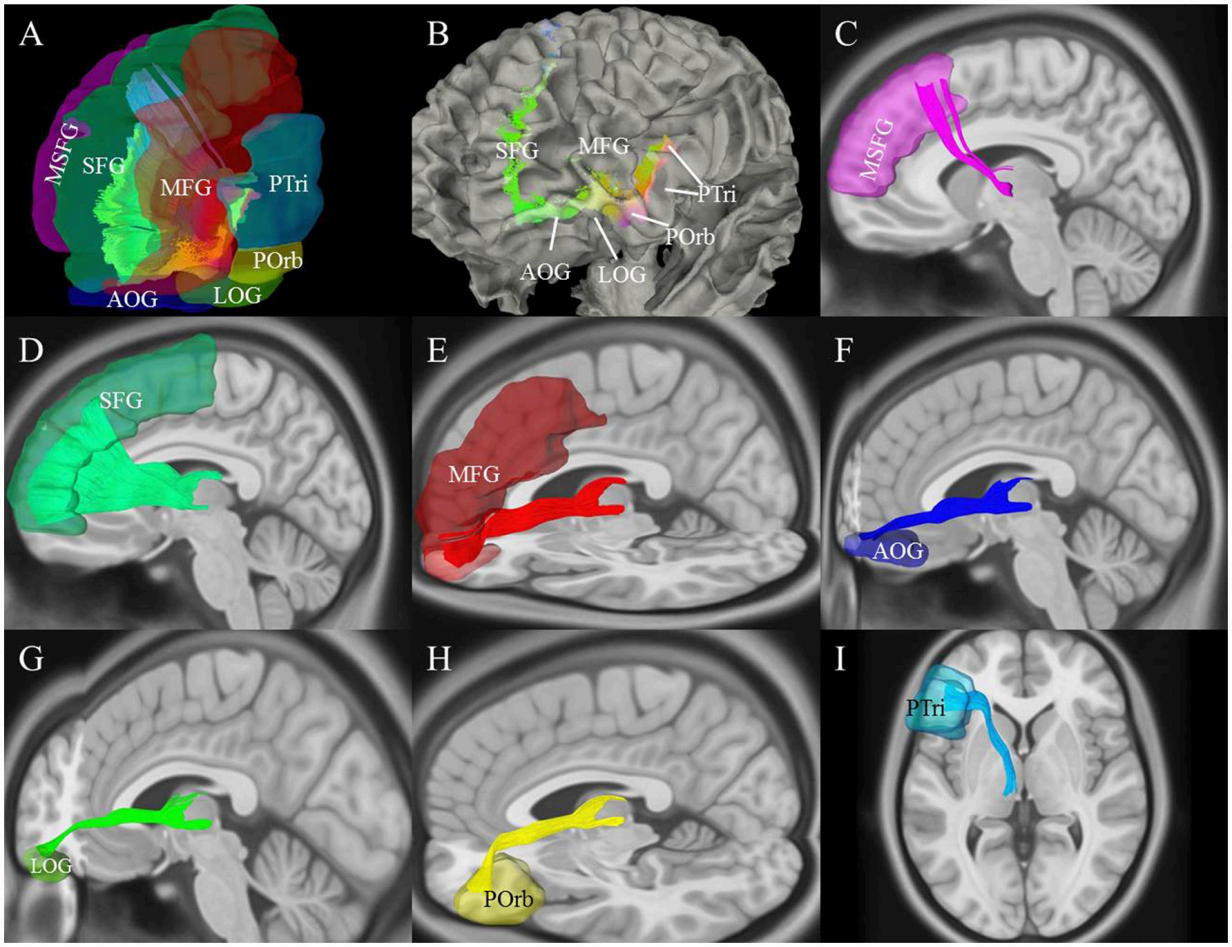
Cortical connectivity and segmentation of the thalamic-prefrontal peduncle (TPP) based on the AAL2 atlas (HCP-842 template). **(A)** The TPP-connected areas in the prefrontal cortex included the SFG, MSFG, MFG, Ptri, Porb, LOG, and AOG. **(B)** Anterior-lateral view: Endpoints of the TPP were overlaid on the white-matter surface. **(C–I)** Views of each tract based on the segmentation. Fibers of each segmentation are colored according to **(A)**. MSFG, medial superior frontal gyrus; SFG, superior frontal gyrus; MFG, middle frontal gyrus; PTri, pars triangularis; POrb, pars orbitalis; AOG, anterior orbital gyrus; LOG, and lateral orbital gyrus.

We used the AAL2 atlas to observe TPP segmentation and connectivity patterns as an alternative approach to understanding the anatomical regions of TPP fiber termination (Figure 6). Seven different TPP termination areas in the prefrontal cortical region were identified: the SFG, medial SFG (MSFG), middle frontal gyrus (MFG), pars triangularis (PTri), pars orbitalis (POrb), anterior orbital gyrus (AOG), and lateral orbital gyrus (LOG). The anterior view of the TPP endpoints has the appearance of the letter “L” or a “fish hook” (Figures 2C,D, 6B). A small bundle of fibers terminated at the caudal MSFG, and a large bundle connected with the middle and rostral SFG. The TPP endpoints were located laterally to the OFC (i.e., AOG and LOG), and in the IFG (i.e., POrb and PTri) and rostral MFG (Figure 6).

**Figure 7:**
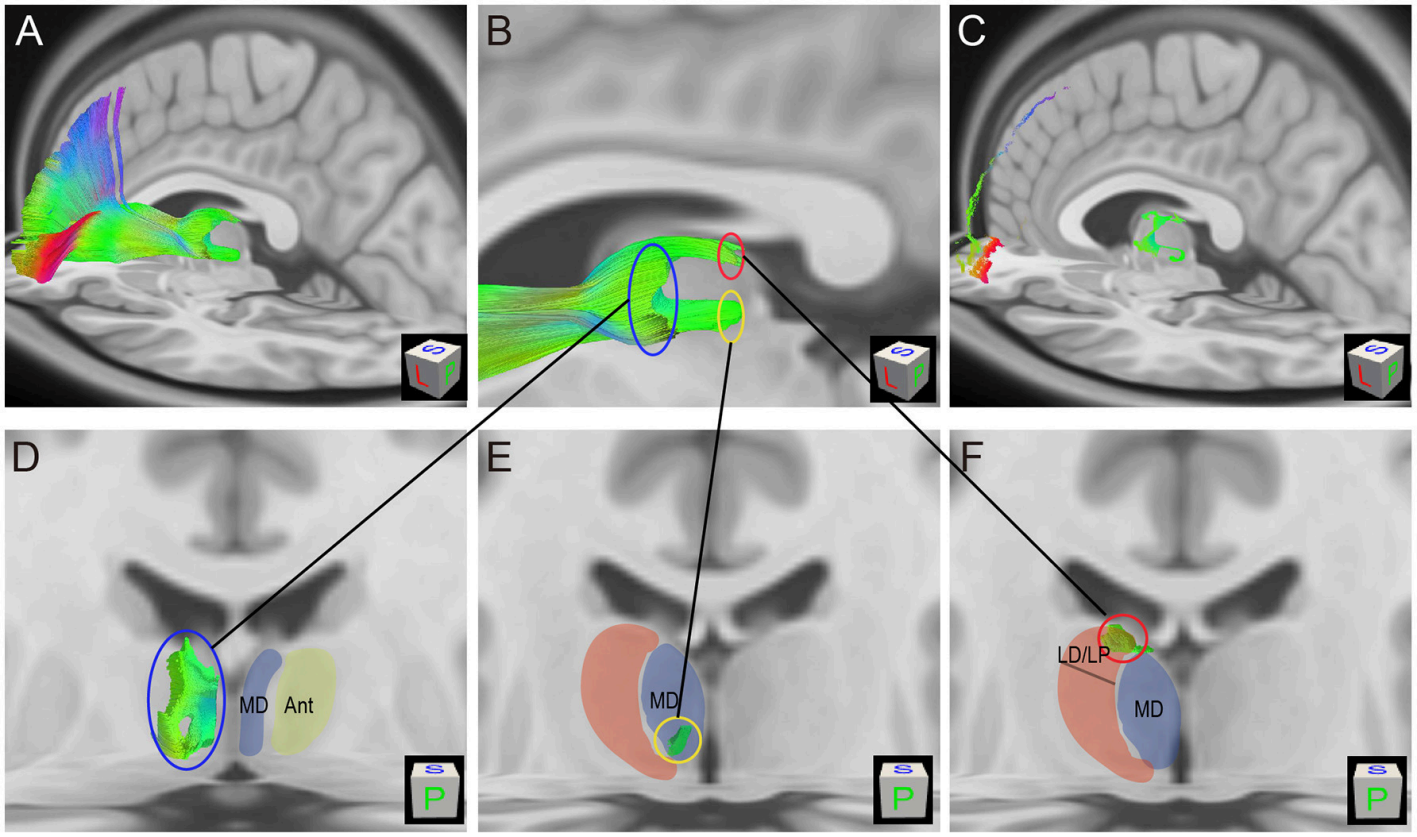
Thalamic-prefrontal peduncle (TPP) fiber origin/termination areas in the thalamus (HCP-842 template). **(A)** Lateral-posterior-superior view of the left TPP fibers. Fibers are overlaid on sagitial and axial T1 MRI slices. **(B)** The origin/termination areas in the thalamus are separated into three parts: dorsal (red circle), ventral (yellow circle), and anterior (blue circle). **(C)** The endpoints of TPP fibers are overlaid on T1 MRI slices. **(D)** The anterior origin/termination area of TPP fibers in the thalamus corresponds to the medial (MD) and anterior (Ant) nuclei of the thalamus. **(E)** The ventral origin/termination area corresponds to the ventral-posterior part of the MD. **(F)** The dorsal origin/termination area of TPP fibers in the thalamus corresponds to the lateral dorsal/lateral posterior nuclei (LD/LP).

**Figure 8:**
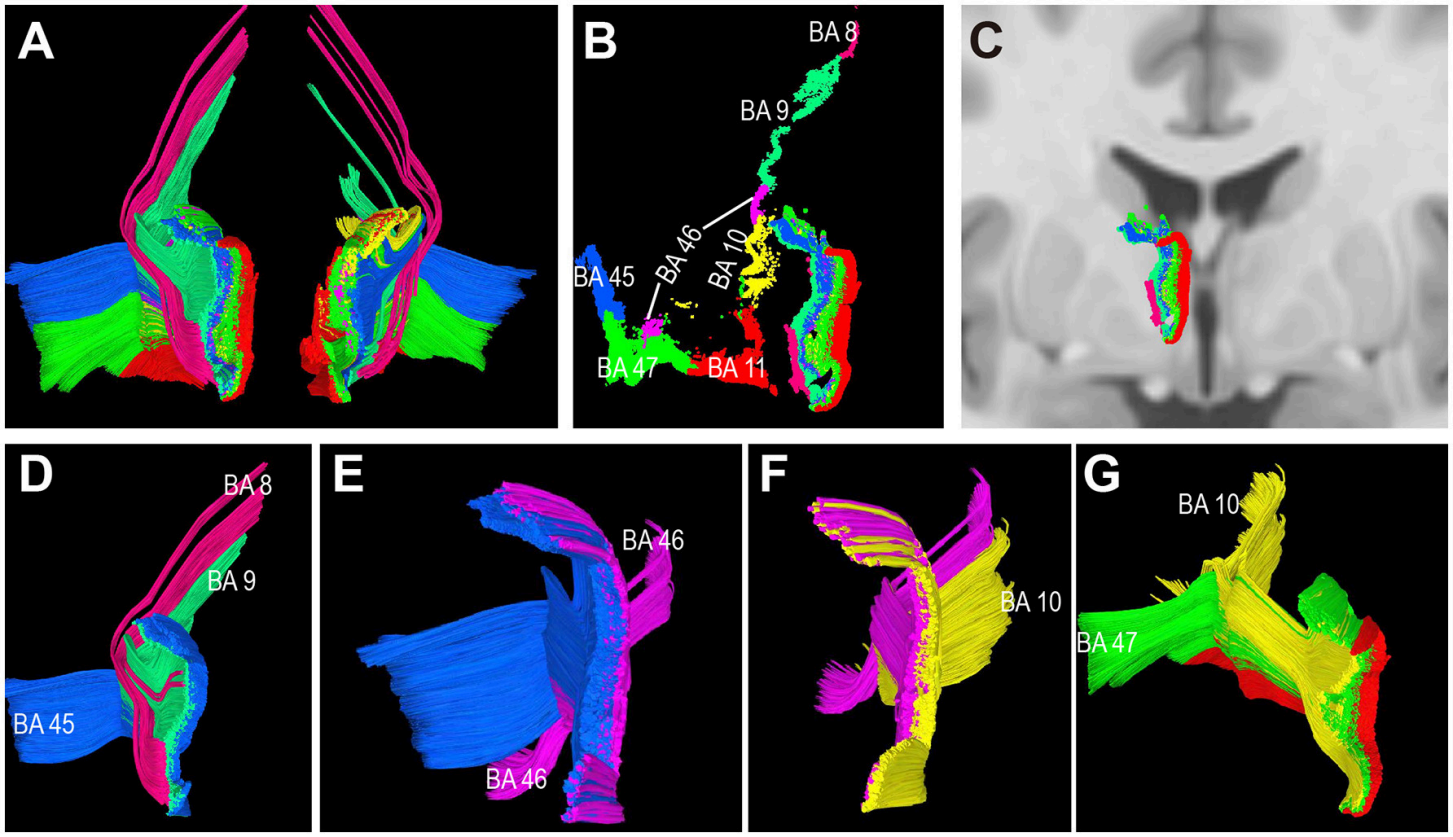
Connectivity patterns of the human thalamic-prefrontal peduncle (TPP) fibers (HCP-842 template). Posterior view: **(A)** Tracts of the left and right TPP. Fibers are assigned different colors based on the different prefrontal Brodmann cortical areas. **(B)** Endpoints of the left TPP show the connectivity patterns of the human TPP. TPP fibers connected the prefrontal cortex with the thalamus layer by layer. Subtracts terminate in the thalamus sequentially as follows: (from medial to lateral) BA 11, BA 47, BA10, BA 46, BA 45, BA9, and BA8. **(C)** The left TPP endpoints in the thalamus are overlaid on T1 coronal MRI slices. **(D–G)** Spatial relationship among subtracts. **(D)** BA 8 tract is located at the most lateral part of the TPP; th eBA 9 tract runs medially to the BA 8 tract; the BA 45 tract is located medial-ventrally to the BA9 tract. **(E)** The BA 46 tract runs medially to the BA 45 tract and splits into two parts (dorsal and ventral tracts). **(F)** The BA 10 tract runs medially to the BA 46 tract. **(G)** The BA 47 tract runs between the BA 11 and BA 10 tracts, then turns laterally to the cortex-crossing part of the BA 10 tract.

To characterize the TPP origin/termination areas in the thalamus, the TPP fibers and their endpoints were overlaid with thalamic T1 MRI slices (Figure 7). The origin/termination areas in the thalamus were separated into three parts: anterior, ventral, and dorsal (Figure 7B). These parts correspond to the medial (MD) and anterior (Ant) nuclei, ventral-posterior part of the MD, and lateral dorsal/lateral posterior nuclei (LD/LP), respectively (Figures 7D–F). To analyze the connectivity patterns of the human TPP fibers, fibers were separated into several subtracts based on the prefronta cortical BAs involved (Figure 8). The left TPP endpoints showed TPP tract connections with the prefrontal cortex and thalamus layer by layer. The location sequence of the subtracts in the thalamus was as follows (from medial to lateral): BA 11, BA 47, BA10, BA 46, BA 45, BA9, and BA8 (Figures 8B,C). The BA 8 tract was located at the most lateral part of the TPP, the BA 9 tract ran medially to the BA 8 tract, and the BA 45 tract was located medial-ventrally to the BA9 tract. The BA 46 tract ran medially to the BA 45 tract and split into two parts (dorsal and ventral). The BA 10 tract ran medially to the BA 46 tract. The BA 47 tract ran between the BA 11 and BA 10 tracts, turning laterally to the cortex-crossing part of the BA 10 tract (Figures 8D–F).

### Quantitative study of the TPP

The TPP volumes in 9 subjects and the HCP-842 template are presented in Table 1. The results were similar between the subject-specific and template-based approaches. Statistical analysis of the subject-specific approach revealed a significant difference between the left and right TPP in tract volume (*P* = 0.03). However, when subcomponents of the TPP were analyzed, only BA 9 showed a significant difference between the left and right hemispheres (*P* = 0.048). The other subcomponents showed no significant differences between the left and right hemispheres (*P* > 0.05) (Table 2).

**Table 2:** The statistical analysis data of the volume of the TPP and its subcomponents.

	**Pairwise difference**					**t**	**df**	***P***
	**Mean value**	***SD***	**Standard error**	**95% confidence interval**				
				**Lower limit**	**Upper limit**			
L—R	5.26	5.98	1.99	0.66	9.86	2.64	8	0.030
L-BA 8—R-BA 8	−0.42	1.28	0.43	−1.41	0.56	−0.99	8	0.349
L-BA 9—R-BA 9	0.89	1.14	0.38	0.01	1.76	2.34	8	0.048
L-BA 10—R-BA 10	0.63	3.02	1.01	−1.69	2.95	0.62	8	0.551
L-BA 11—R-BA 11	0.63	3.30	1.10	−1.91	3.17	0.57	8	0.582
L-BA 45—R-BA 45	1.75	3.11	1.04	−0.64	4.14	1.69	8	0.129
L-BA 46—R-BA 46	0.62	2.64	0.88	−1.41	2.65	0.71	8	0.498
L-BA 47—R-BA 47	1.07	2.88	0.96	−1.15	3.28	1.11	8	0.299

## Discussion

Previous studies have delineated the TPP fibers in monkey and human brains using different methods, including fiber microdissection, autoradiography, and diffusion imaging studies. However, these reports do not contain detailed information on the connections between the thalamus and prefrontal cortex. Here, we further characterize the human TPP *in vivo* using the DSI technique. We also report the fiber trajectories, spatial relationships, cortical connectivity, segmentation, and a quantitative assessment of the human TPP.

We analyzed the TPP using two approaches, subject-specific and template-based, to enhance the reliability of our results. The TPP connections presented here were first observed in 9 subjects (18 hemispheres) and confirmed by an HCP-842 template, the average of 842 subjects' HCP data. Using this template, we obtained results from a large sample simultaneously. Unlike the subject-specific approach, the template approach does not allow for the assessment of individual differences. However, averaging data from a larger sample does provide unique information on TPP connectivity patterns. Significantly, the two approaches revealed similar connectivity patterns in the present study.

Descriptions of the thalamic peduncles date back to a century ago, when Charles Judson Herrick first delineated the fibers connecting the thalamus and cortex (Herrick, 1915). Autoradiographic technology, the “gold standard” of imaging, enabled the study of white matter fiber connectivity. An autoradiography study of the monkey brain described in detail the thalamic peduncles, and showed that all regions of the prefrontal cortex sent fibers to the thalamus via the anterior limb of the internal capsule to form the ATP, whereas fibers from the orbital cortex connected to the thalamus through the ITP (Schmahmann and Pandya, 2006). However, recent evidence, such as a report on the middle longitudinal fascicle (Wang et al., 2013), appeared to show that the white matter fiber pathways in humans and monkeys were partially different. Because the present study was based on the human brain *in vivo*, our results are likely to be more reliable. Previous fiber microdissection studies have identified the TPP trajectory directly (de Castro et al., 2005; Serra et al., 2017), showing that the TPP fibers originating from the thalamus radiate anteriorly and constitute part of the anterior limb of the internal capsule. Studies comparing DSI with classic fiber microdissection and autoradiography techniques have shown DSI to be in good agreement with the other methods both in human and monkey brain white matter (Schmahmann et al., 2007; Fernandez-Miranda et al., 2012; Wang et al., 2013; Fernández-Miranda et al., 2015). However, because the thalamocortical fibers, which join into the internal capsule, are difficult to distinguish from the other fibers by microdissection, the fiber dissection studies may not precisely describe the continuous fiber trajectories from the thalamus to brain cortex. Because we utilized the DSI technique, which can sufficiently resolve the complex fiber crossings, we were able to track the fibers from the thalamus to prefrontal cortex continuously, and to identify the termination areas of the TPP fibers in the prefrontal cortex. Our results show that the TPP fibers project anteriorly from the thalamus toward the prefrontal lobe along the anterior limb of the internal capsule. The connected areas in the prefrontal cortex are BAs 8, 9, 10, 11, 45, 46, and 47, and can alternatively be described as AAL2 areas MSFG, SFG, MFG, PTri, POrb, AOG, and LOG, respectively. Previous studies using diffusion imaging techniques, including DTI and HDFT (Behrens et al., 2003; Wakana et al., 2004; Fernandez-Miranda et al., 2012; Nishio et al., 2014), have shown fibers connecting the prefrontal region with thalamus without revealing a specific TPP connectivity pattern.

DTI is unable to solve the fiber crossing problem and identify fiber origins and destinations. The previous DTI reports have only described fan-like fibers projecting to the SFG. Because of the influence of the crossing fibers, such as in the A-CC, TPP fiber trajectories tracked by DTI may not include all cortical connections. Because DSI represents a better method of fiber tracking, we were able to characterize the spatial relationship of the TPP with adjacent fiber tracts (Cort-Sp, IFOF, and A-CC) (Figure 4). HDFT studies have shown the origins/terminations of the thalamic peduncles: the fibers originating from the orbital-frontal region are linked to the most medial and anterior portions of the thalamus, those from the prefrontal area to the ventral anterior and lateral regions of the thalamus, central lobule fibers to the ventral posterior zone, and parietal-occipital fibers to the posterior and inferior zones of the thalamus (Fernandez-Miranda et al., 2012). Our study describes the origin/termination areas of the TPP based on the thalamic structure. The TPP links to the Ant, MD, and LD/LP portions of the thalamus, similar to the HDFT studies and in agreement with neurohistological studies (Nieuwenhuys et al., 2008; Fernandez-Miranda et al., 2012). While DTI, HDFT, and DSI utilize deterministic tractography algorithms, a study of the connections between the human thalamus and cortex based on a probabilistic approach showed that the fibers linked the prefrontal cortex with the MD, ventral anterior (VA), and parts of the anterior portion of the thalamus (Behrens et al., 2003), largely in agreement with our results. A functional MRI study also showed functional connectivity between the prefrontal cortex and mediodorsal and anterior nuclear areas of the thalamus, similar to our results (Zhang et al., 2008).

Although the thalamic peduncles have been characterized in detail, their precise cortical connections with the thalamus have remained unclear. For comparison and a better understanding of the connections, we used two prefrontal cortical maps (from the BA and AAL atlases) to describe the precise TPP connections with the region and discovered a novel TPP connectivity pattern. Our results show that the TPP fibers connect the prefrontal cortex with the thalamus layer by layer based on the prefrontal BA atlas. The tract which connects the orbital-frontal area (BA11) with the thalamus is located at the most medial portion of the TPP, the SFG tract (BA8, 9) at the most lateral part of the TPP, and the tracts projecting to the IFG at the middle of the TPP (Figure 8). These results were obtained using both the subject-specific and template approaches.

Studies of thalamic function indicate that the thalamus is a relay center for sensory and motor information, awareness, attention, and other cognitive processes such as memory and language (Herrero et al., 2002; de Bourbon-Teles et al., 2014; Dalrymple-Alford et al., 2015). The function of the prefrontal cortex has been researched extensively and includes cognitive abilities, social emotion, executive functioning, motor control, and language (Teffer and Semendeferi, 2012; Nestor et al., 2013). Because the TPP is a large bundle of fibers that connects two important brain regions, the thalamus and prefrontal cortex, it may play a significant role in the functions mentioned above. Interestingly, we found TPP fibers extending to BA45 (PTri), which corresponds to the anterior portion of Broca's area, commonly considered to be the substrate of functional language processing. The brain cortical functions involve white matter fibers; thus, our findings may provide strong new evidence for the role of the thalamus in language function. In addition to BA45, other large bundles of fibers terminated in the OFC, SFG, and BA47. However, only a small fiber bundle terminated at the very anterior portion of the MFG. Given this pattern of TPP connectivity, we can focus our attention on the correlated areas in future investigations of TPP fiber functions and the relationship between the thalamus and cortex.

Although the DSI technique we employed is better able to assess complex fibers, such as crossing fibers, than is DTI, it still has limitations. For example, when analyzing the TPP, we found some false continuations of fibers at the lateral edge of the TPP; these fibers did not terminate in the thalamus, which should be part of the Cort-Sp. Our study only focused on the connectivity between the prefrontal cortex and thalamus, we may loss some fibers information, especially in the region near the parietal cortex. However, these limitations did not influence the TPP connectivity patterns eventually identified. Previous studies comparing the results of DSI analyses and those of fiber dissections have shown that the former produces more reliable evidence (Fernandez-Miranda et al., 2012; Wang et al., 2013; Fernández-Miranda et al., 2015). Therefore, the results of the present study are likely to be reliable and may provide a foundation for future studies of the thalamic peduncles.

## Conclusions

We studied the TPP connectivity patterns in the human brain *in vivo* using both subject-specific and template-based approaches. Detailed connections between the thalamus and prefrontal cortex were described. Our results may further the study of the functions of the thalamus and prefrontal cortex based on fiber connectivity patterns.

## Author contributions

Conceived and designed the experiments: YiW and CS. Performed the experiments: CS, CW, YB, and YoW. Statistical analysis: RC and XL. Data interpretation and figure preparation: CS and YiW. Wrote and revised the manuscript: CS, YoW, and YiW.

### Conflict of interest statement

The authors declare that the research was conducted in the absence of any commercial or financial relationships that could be construed as a potential conflict of interest.
